# Symptoms of Depression and Anxiety Among Elite High School Student-Athletes in Sweden During the COVID-19 Pandemic: A Longitudinal Study

**DOI:** 10.1192/j.eurpsy.2023.1240

**Published:** 2023-07-19

**Authors:** M. J. Andersson, G. Kenttä, K. Moesch, E. Borg, E. Claesdotter-Knutsson, A. Håkansson

**Affiliations:** 1 Department of Clinical Sciences, Lund University, Lund; 2 The Swedish School of Sport and Health Sciences, Stockholm; 3 Department of Sports Science, Malmö University, Malmö; 4 Department of Psychology - Perception and Psychophysics, Stockholm University, Stockholm, Sweden

## Abstract

**Introduction:**

The COVID-19 pandemic precipitated numerous changes in daily life, including the cancellation and restriction of sports trainings and competitions globally. Because engagement in sports contributes positively to the physical and psychosocial development of adolescents, restricting these activities may have led to long-term changes in mental health, especially among high school student-athletes that spend a significant amount of time training and competing.

**Objectives:**

We sought to (1) compare overall prevalence rates and symptom severity of depression and anxiety between 2021 and 2022, (2) assess cohort and class-level differences on internalizing measures, and (3) identify demographic and health risk factors for developing depressive and anxiety symptoms in 2022 and compare the composition of these models predicting depression and anxiety with those proposed by Håkansson et al. (Front. sports act. living 2022; 4 943402) on student-athletes in the 2021 sample.

**Methods:**

Using a cross-sectional study design with repeated measures, we measured rates of depression using the Patient Health Questionnaire-2 scale (PHQ-2) and anxiety using the Generalized Anxiety Disorder-2 scale (GAD-2) in student-athletes attending elite sport high schools in Sweden during the second wave of the pandemic (February 2021) and after all restrictions were lifted (February 2022).

**Results:**

As illustrated in Table 1, the overall prevalence of depression among student-athletes declined significantly from 19.8% in 2021 to 17.8% in 2022, whereas the percentage of student-athletes screening for anxiety did not change significantly (17.4% in 2021 to 18.4% in 2022).

**Table 1:** Depression and Anxiety Measures 2021-2022

Variable	2021	2022	*p_raw_*	*p_adj_*
Positive diagnosis, *n* (%) *^a^*				
PHQ-2 ≥ 3	1390 (19.8%)	1107 (17.8%)	.002	.007
GAD-2 ≥ 3	1219 (17.4%)	1147 (18.4%)	.125	.187
Symptom measure *M* (*SD*) *^b^*				
PHQ-2 [0-6]	1.38 (1.52)	1.25 (1.48)	< .001	< .001
GAD-2 [0-6]	1.35 (1.57)	1.36 (1.59)	.784	.840

*Note.* Comparison of psychological health measures across years. *P*-values adjusted for multiple comparison using Benjamini & Hochberg (1995)^36^ procedure.

a Chi-square test of homogeneity

b independent-samples *t*-test

Cohort-level analyses revealed older students exhibited decreases in depressive symptoms (Figure 1), while younger cohorts experienced increases in symptoms of anxiety (Image 2) from 2021 to 2022. Logistic regressions revealed that being female, reporting poorer mental health due to COVID-19, and excessive worry over one’s career in sports were significant predictors of both depression and anxiety screenings in the 2022 sample (Image 2).

**Image:**

**Figure:**
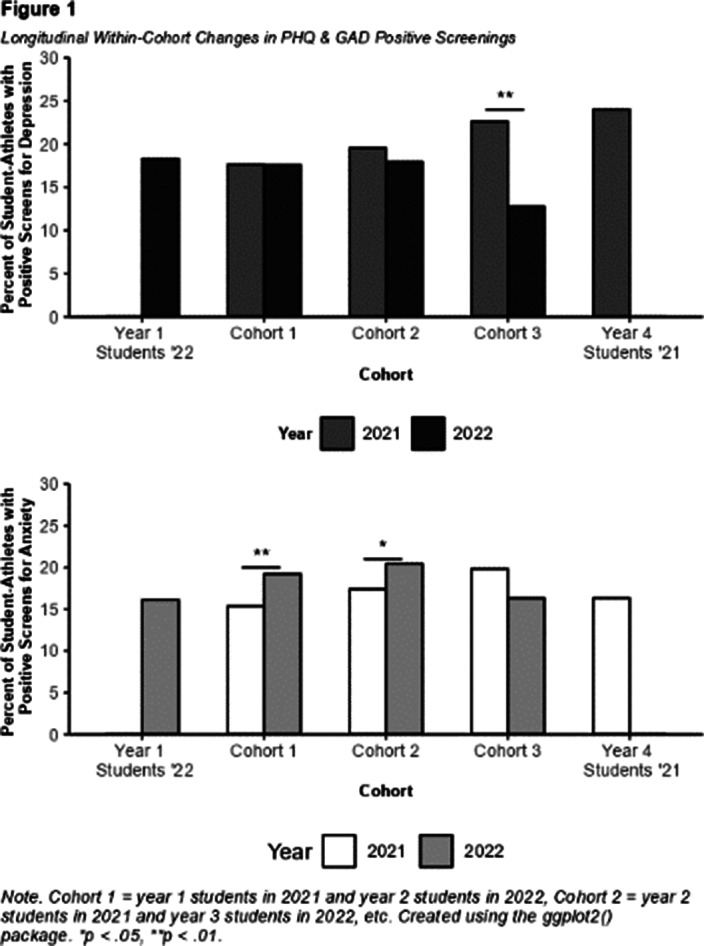

**Image 2:**

**Figure:**
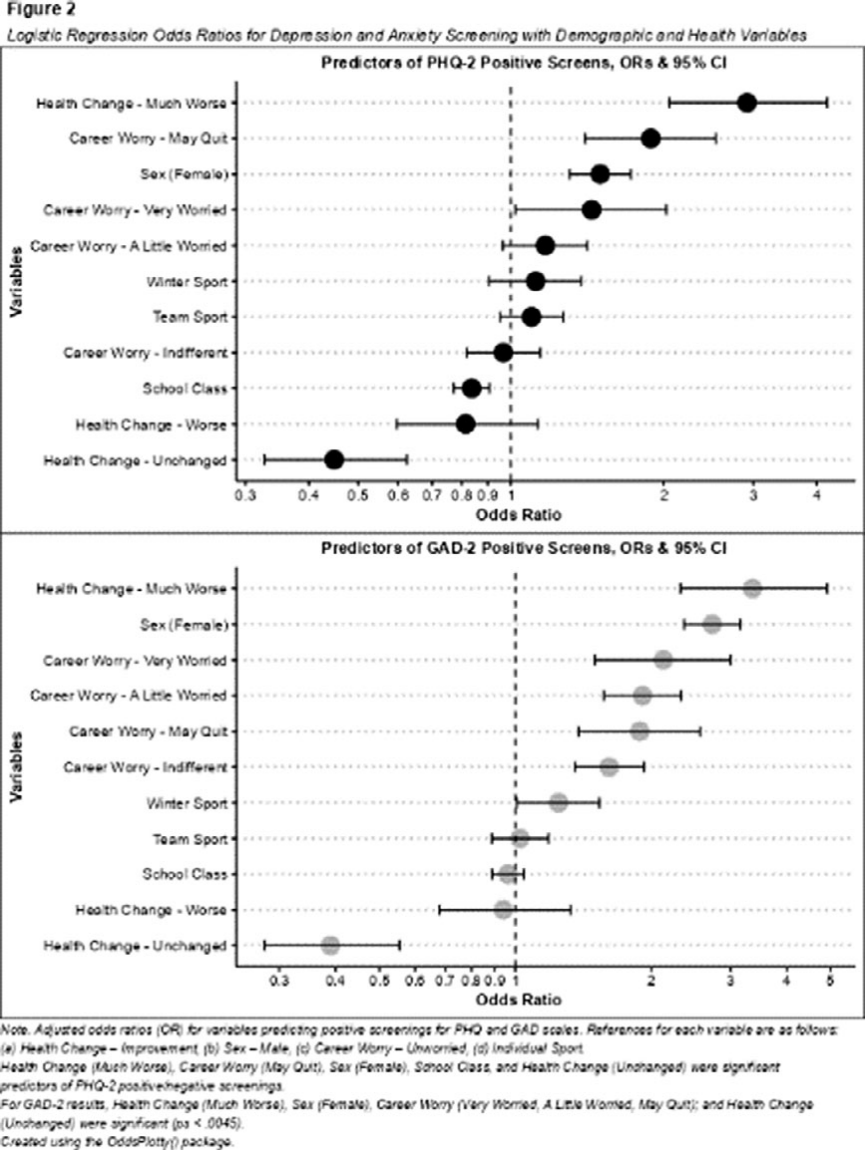

**Conclusions:**

In comparison to periods when sports participation was limited in February 2021, the lifting of restrictions in February 2022 was associated with overall reduced levels of depression, but not anxiety.

**Disclosure of Interest:**

M. Andersson: None Declared, G. Kenttä: None Declared, K. Moesch: None Declared, E. Borg: None Declared, E. Claesdotter-Knutsson: None Declared, A. Håkansson Grant / Research support from: AH receives financing from the Swedish state-owned gambling operator, AB Svenska Spel, and the state-owned alcohol monopoly. Neither were involved in the study planning, execution, or decision to publish the current article.

